# An Overview of Waste Milk Feeding Effect on Growth Performance, Metabolism, Antioxidant Status and Immunity of Dairy Calves

**DOI:** 10.3389/fvets.2022.898295

**Published:** 2022-05-17

**Authors:** Yulin Ma, Muhammad Zahoor Khan, Jianxin Xiao, Gibson Maswayi Alugongo, Xu Chen, Shengli Li, Yajing Wang, Zhijun Cao

**Affiliations:** ^1^State Key Laboratory of Animal Nutrition, College of Animal Science and Technology, China Agricultural University, Beijing, China; ^2^University of Agriculture, Dera Ismail Khan, Khyber Pakhtunkhwa, Pakistan

**Keywords:** waste milk, microbiota, antibiotic residues, pasteurization, growth performance, rumen fermentation

## Abstract

Waste milk (WM) is a part of the milk produced on dairy farms, which is usually unsuitable for human consumption. The WM contains transition milk, mastitis milk, colostrum, milk with somatic cells, blood ***(****Hemolactia****)*, **harmful pathogens, pathogenic and antibiotic residues. Due to the high cost of milk replacer (MR), dairy farmers prefer raw WM to feed their calves. It has been well established that WM has a greater nutritive value than MR. Hence WM can contribute to improved growth, rumen development, and immune-associated parameters when fed to dairy calves. However, feeding raw WM before weaning has continuously raised some critical concerns. The pathogenic load and antibiotic residues in raw WM may increase the risk of diseases and antibacterial resistance in calves. Thus, pasteurization has been recommended as an effective method to decrease the risk of diseases in calves by killing/inhibiting the pathogenic microorganisms in the raw WM. Altogether, the current review provides a brief overview of the interplay between the positive role of raw WM in the overall performance of dairy calves, limitations of raw WM as a feed source and how to overcome these issues arising from feeding raw WM.

## Introduction

The productive efficiency of dairy farms is determined by many factors, including good management of dairy calves. There are no returns for the dairy farm in the first 2 years of rearing heifers, with average costs for raising a Holstein heifer estimated at $1,225 in the US (1, 2). It has been suggested that these costs are much higher in China, with estimates projected to be almost 1.34 times those of the US (3).

On most Chinese dairy farms, calves are weaned at least 2–3 months after birth (4). A calf is most likely to have consumed ~350–500 kg of standard milk (SM) or milk replacer (MR) during that period. Therefore, in production systems that rely solely on feeding raw SM, the rearing costs might be greater because of the opportunity cost involved; SM is the sole income source. Hence, several feeding techniques have been explored in the past few decades to minimize calf rearing costs and ultimately reduce farm costs during the non-productive period.

Waste milk (WM) has gotten considerable attention among dairy farmers because of its perceived low cost and desirable nutritive value. All non-saleable milk, including colostrum, transition milk, mastitis milk, colostrum, milk with somatic cells, blood (Hemolactia), harmful pathogens and pathogenic and antibiotic residues, is usually graded as raw WM (5). Mei et al. (6) reported that the raw WM accounts for about 2–4 percent of China's total milk production, equating to 0.8–1.6 million tons of milk produced annually in China. These significant amounts of milk represent a loss of income for the dairy farm and can also be an environmental hazard. However, since some dairy farms can have a significant amount of raw WM with a nutrient profile as good as that of salable raw SM, farms dispose of the milk by feeding it to dairy calves (7). For example, a study from the United States reported that raw WM was the main dietary liquid feed fed to 40.1%, while MR was fed to slightly <35.0% of calves surveyed (8). Although feeding WM to calves is considered cost-effective, its use on dairy farms remains controversial (9).

**Figure 1:**
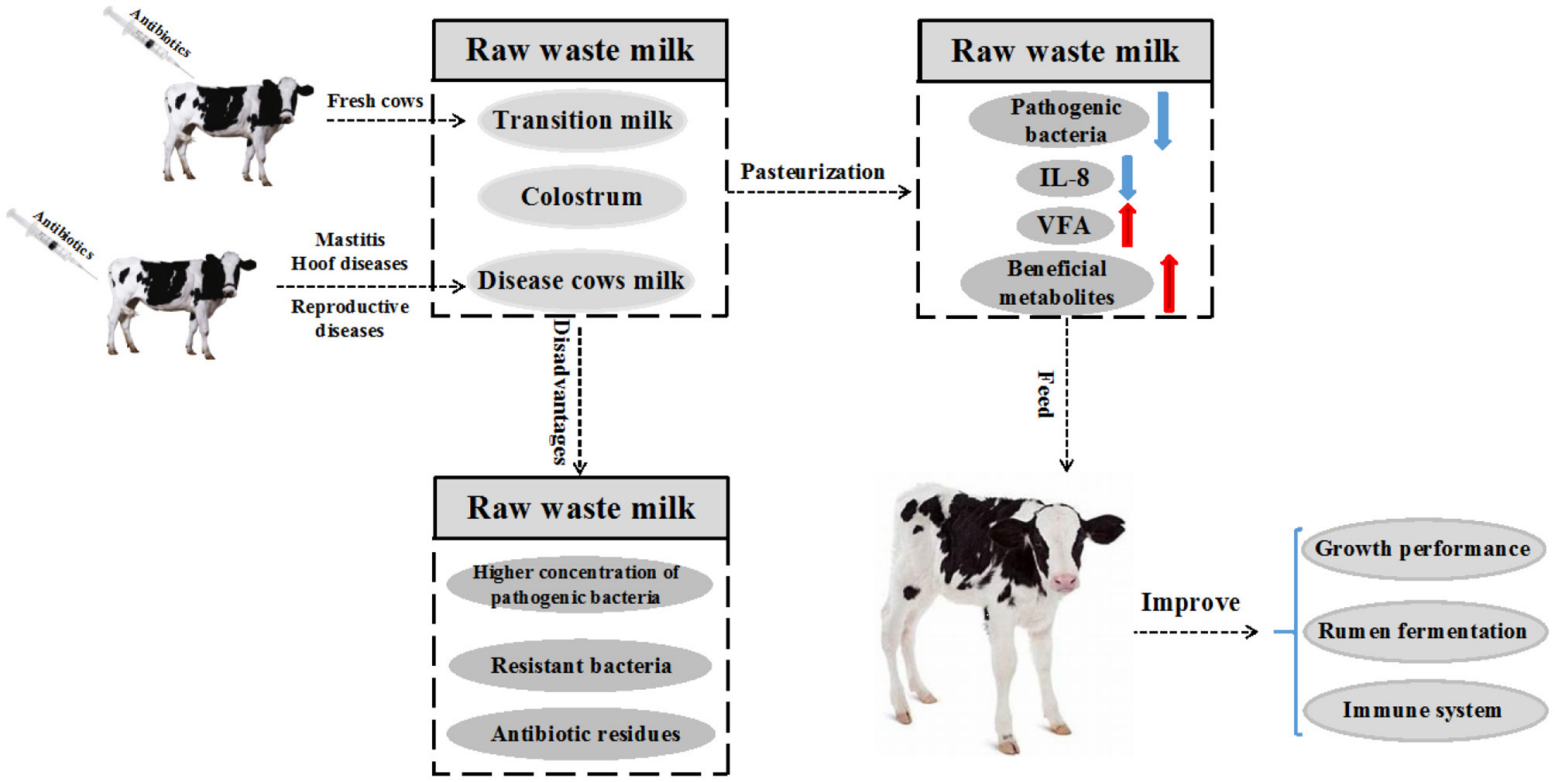
Raw waste milk (WM) refers to milk that cannot be sold to the market for human consumption. The milk is obtained mainly from fresh cows and diseased cows under treatment with antibiotics or whose antibiotic withdrawal period has not elapsed, resulting in very low concentrations of these antibiotics being passed through milk. Pasteurization is often performed before feeding WM to reduce health risks in calves. Pasteurized WM reduces the number of harmful bacteria, IL-8 and increases the concentration of beneficial metabolites and volatile fatty acids (VFA). Finally, the growth performance, rumen fermentation and immune system of the calves are improved.

Raw WM contains a high concentration of viable bacteria and antibiotic residues (10), which are linked to an increased incidence of diarrhea in calves (11). It has been reported that *Salmonella* spp. and *Mycoplasma* spp. in raw WM are among the major pathogenic bacteria that pose a threat to calf health (12). Consequently, some studies have documented that feeding raw WM might contribute to the development of antimicrobial-resistant bacteria in the gut of calves (13, 14). Indeed, a shift in the prevalence of antimicrobial resistance has been observed in calves fed raw WM (15, 16). Furthermore, the bovine viral diarrhea virus (BVDV) is an important global pathogen in the livestock industry (17). It was recently reported that feeding calves with milk from lactating cows carrying BVDV resulted in serious health issues (18). Proper treatment of raw WM is highly recommended to overcome these health challenges. Pasteurization has been promoted as a viable option for treating WM and reducing levels of harmful bacteria in milk to acceptable limits (19). It is plausible that feeding calves with pasteurized raw WM could cost ($0.69/day) less to raise than those fed commercial MR, even when the cost of purchasing a pasteurizer is included (9). Moreover, calves fed pasteurized waste milk (PWM) showed increased growth and had lower morbidity and mortality during the pre-weaning period (9). Alternatively, organic acids could be used to acidify WM (AWM) and reduce the health risk of feeding raw WM to the calves (20). The effect of raw WM on overall calf performance is summarized in Figure 1. The current review discusses the drawbacks of utilizing raw WM to feed dairy calves. In addition, the impact of pasteurization in reducing bacterial load in milk has also been explored in depth.

## Effect of WM Feeding on Calf Performance

### Effect on Calf Growth Performance

From birth to weaning, dairy calves endure numerous challenges and stressors, including greater morbidity rates (21), which might affect their growth performance. The nutritional and health characteristics of these calves, if not effectively controlled, will lead to increased production costs and environmental hazards. A range of factors such as the quantity and quality of liquid feed, environmental changes, feeding methods, and solid feed intake are critical to calf growth and health (22).

Calves fed raw WM had greater average daily gain and serum total protein, albumin, total cholesterol, high-density lipoprotein, triglycerides, growth hormone, immunoglobulin (Ig) A and IgM concentrations compared to raw SM (23), which could be attributed to the high amount of milk fat and solid non-fat percentages, or presence of antibiotic residues in raw WM (5). Similar studies showed that calves fed raw WM gained more body weight than calves fed MR or SM (5, 23–25). One probable mechanism is the growth-promoting effects of raw WM, whereby the antibiotic residues present in the raw WM alters the intestinal flora resulting in a healthy intestinal environment (26). Subsequently, the calves direct the extra energy available toward growth rather than fueling the immune function while trying to counteract the pathogen and antigens in the gut lumen (27).

The range of penicillin concentrations utilized in a dose-regulated experiment was determined using the concentration of β-lactam antibiotic residues discovered in milk from treated cows (13). In addition, they also reported that the resistance of gut bacteria to antibiotics increased with the supplementation of raw WM in calves having higher concentrations of penicillin in the milk (13).

The effect of substituting PWM with raw SM for 3 weeks on dairy calves' growth and health performance has been well documented (28). It has consistently been reported that calves fed PWM gained more BW than those fed raw SM during the post-weaning and overall periods. In the pre-weaning, post-weaning, and overall periods, calves fed PWM had higher average daily gains than those fed raw SM. Moreover, feed efficiency and health were improved with feeding the PWM during all the studied periods. A study reported that calves fed PSM improved daily gain (29).

Unlike raw SM, the effect of MR on calf growth performance depends on its composition. Hence, studies that have compared raw SM or PWM and MR have shown inconsistent results. It is well established that source of protein in milk replacer can interfere with growth, with some early studies reporting that calves fed milk-derived protein MR performed better than those fed soybean protein concentrate as the main protein source (30). On the contrary, calves fed raw SM and MR had similar growth performance, though the MR contained plant protein (31). Additionally, an optimal protein level in MR is critical to support growth and reduce environmental pollution. Calves fed an MR containing 22% crude protein had better growth performance and nutrient utilization than animals fed either 18 or 26% of crude protein on a DM basis (32).

Alternatively, AWM could be fed to promote calf growth (33). Adding an organic acid to milk decreases the WM pH and diarrhea and increases the daily gain in dairy calves (23). The AWM might have an improved flavor, which promotes appetite in calves (34). Presently economic considerations generally hinder the use of raw SM on dairy farms. Partial replacement of raw SM with raw WM is recommended in dairy calves at an early stage of life to maintain a maximum growth rate and health benefits (9). A summary of the effects of raw WM and PWM on calf growth performance is shown in Table 1.

**Table 1:** Effect of feeding raw WM and PWM on the growth performance of calves.

**Feed type**	**Types/amount of antibiotics**	**Feeding groups**	**Data**	**Growth performance**	**References**
WM	0.025 mg/L of streptomycin, 0.1 mg/L of tetracycline, 0.024 mg/L of penicillin, and 0.33 mg/L of ceftiofur	PWM and raw WM	Raw WM: 733 g/d PWM:798 g/d	No adverse effect on average daily gain.	(28)
WM	Chlortetracycline, oxytetracycline, tylosin, and monensin	Raw WM and MR	MR: 450 g/d PWM: 650 g/d	Increased average daily gain of calves	(35)
WM	ND; Milk from cows treated with veterinary drugs due to mastitis or other diseases	Raw SM and WM	Raw SM: 258 g/d Raw WM: 525 g/d	Increased average daily gain of calves	(23)
WM	Amoxicillin/ clavulanic acid, enrofloxacin, cefquinome, ceftiofur, tylosin, oxytetracycline and penethamate hydroio dide.	Raw SM and WM	Raw WM: 587 g/d Raw SM: 538 g/d	There was no unusual effect on average daily gain.	(5)
WM	ND; Milk from cows treated with veterinary drugs due to diseases	Raw SM and WM	Raw SM: 690 g/d Raw WM: 730 g/d	No negative effect was on average daily gain.	(36)
WM	ND; Milk from cows with clinical mastitis, placental retention, metritis, or foot infections	Raw SM and WM	Raw SM: 670 g/d Raw WM: 710 g/d	There is no adverse effect on average daily gain.	(37)
PWM	Mainly cephalosporins and lincosamides	PWM and Raw WM	PWM: 780 g/d Raw WM: 670 g/d	Increased average daily gain of calves	(38)
PWM	ND; Milk from cows treated with veterinary drugs due to mastitis or other diseases	PWM and Raw SM	PWM: 454 g/d Raw WM: 258 g/d	Improved the average daily gain of calves	(23)
PWM	Per 1 ml contains 100,000 U.I. penicillin G procaine, 100,000 U.I. penicillin G benzathine, 120 mg dihidroestreptomycin sulfate, and 0.2 mg dexamethasone sodium phosphate	PWM and NMR	PWM: 543 g/d Raw WM: 515 g/d	There was no negative average daily gain.	(39)
PWM	Amoxicillin/clavulanic acid, enrofloxacin, cefquinome, ceftiofur, tylosin, oxytetracycline and penethamate hydroio dide.	PWM and Raw SM	PWM: 584 g/d Raw SM: 538 g/d	There was no undesirable effect on average daily gain.	(5)
PWM	ND; Milk from cows with clinical mastitis, placental retention, metritis, or foot infections	PWM, Raw SM and raw WM	PWM: 345 g/d Raw SM: 375 g/d Raw WM: 392 g/d	There was no adverse effect on average daily gain.	(37)

*Pasteurized waste milk (PWM); Waste milk (WM); non-medicated milk replacer containing sodium butyrate and active probiotic Bacillus amyloliquefaciens (NMR); In cases where the antibiotics were not determined (ND), the composition of milk used is stated*.

### Effect on Rumen Fermentation in Dairy Calves

Rumen development relies on the pre-weaning colonization of the gastrointestinal tract with bacteria, which results in the physiological changes and transition from a non-ruminant to a ruminant (40). On the other hand, dietary changes may result in long-term changes in the composition of rumen bacteria (41–43).

Pasteurized waste milk substantially altered the gut microbial composition in pre-weaning calves, especially *Bacteroidetes* spp. and *Firmicutes* spp. (44) which significantly increased in calves fed PWM compared to raw SM. In a different study, calves fed raw WM exhibited a richer rumen microbiota than those fed raw SM (35). The raw WM contains greater milk protein content entering the rumen than raw SM (45) due to its substantial amount of transition milk. The rumen of raw WM-fed calves tends to have a higher concentration of isovalerate, a true reflection of the high protein content in the raw WM. The higher concentration of isovalerate has been associated with increased numbers of *Butyrivibrio fibrisolvens* in calves (46).

In the rumen, microorganisms produce branched-chain volatile fatty acids (VFAs) through decarboxylation of branched-chain amino acids and peroxide deamination (45). The VFAs, especially butyrate, stimulate the growth of the rumen papilla and epithelium and are considered the most critical elements influencing rumen development (47). Calves fed MR plus four antibiotics (0.024 mg/L penicillin, 0.025 mg/L streptomycins, 0.1 mg/L tetracyclines, and 0.33 mg/L ceftiofur) had a reduced and higher richness of *Prevotella* spp. and *Acetitomaculum* spp., respectively, at the genus level. As a result, residual antibiotics administered to raw WM calves enhanced rumen fermentation, ruminal papillae growth, and increased rumen acetic acid production (2). The relative abundance of *Prevotella* spp., one of the most diverse and core flora at the genus level (48), was reduced by antibiotic residues, possibly due to their sensitivity to a penicillin (49) and tetracycline (50) antibiotics. Antibiotics such as monensin, gentamicin, virginithromycin, ampicillin, ceftriazine, penicillin, and oxytetracycline are routinely used to treat mastitis in dairy cows. Chlortetracycline, oxytetracycline, tylosin, and monensin have been demonstrated to prevent rumen bacteria from breaking down cellulose and producing VFA *in vivo* (51). In addition, ampicillin, ceftriazine, penicillin and oxytetracycline inclusion in calf milk can affect the fecal microbial population composition (35, 52). A recent study supported the idea that gentamicin inhibits *Prevotella intermedia* (53). The abundance of *Prevotella* spp. was likewise reduced in calves given raw WM with concentrations of 0.024 mg/L penicillin, 0.025 mg/L streptomycins, 0.10 mg/L tetracycline, and 0.33 mg/L ceftiofur (2). Feeding raw SM has an important effect on calf rumen development. A study reported lower acetate and propionate levels in the rumen of calves fed raw SM compared to PWM (37). A study found that feeding acidified SM to calves within 14 weeks of age was helpful for rumen development (54). Calf rumen pH is an important indicator for assessing rumen development. The optimal pH of calf rumen should be between 5.8 and 6.4 pH units under good feeding practice (55). A study reported that the addition of inulin to raw SM can influence the pH of the rumen by making it more alkaline, and can accelerate postnatal rumen development and improve its functionality (56), and adding 12 g of inulin to raw SM when feeding calves could improve rumen papillae development, as seen from increased length and width of the papilla, especially in the Saccus ventralis region. A study in our research group tried to improve rumen development by adding butyrate to raw SM (57). However, raw SM with butyrate had no significant effect on rumen development (57). A study reported that PSM reduced the calf diarrhea rates and improved health compared to SM (58). Likewise, MR can positively affect calf rumen development (59). Interestingly, most studies have focused on the effects of combining milk replacer with other beneficial supplements, such as probiotics and milk replacer, on the calf rumen (60, 61). Indeed, a study found that adding probiotics to MR improved rumen development (61).

### Effect on Intestinal Villi Development

Injury to the intestinal mucosa disrupts the intestinal epithelial barrier function resulting in enteritis and diarrhea in dairy calves (62). Calves fed both raw WM and PWM had uniform villi in the ileum, while those fed raw SM were irregular (23). However, pasteurization seems to further improve the villi distribution and conformity in calves fed PWM compared to raw WM. The high concentration of growth factors and bioactive peptides in PWM might partially explain the superior developed intestinal villi compared to raw WM (63, 64).

## Effect of WM on Intestinal Microbiota and Microbiology in the Intestinal Tract

### Effect on Intestinal Microbiota in Calves

Shortly after birth, different bacteria colonize the calf's gastrointestinal system (27, 65). The composition and activity of these microbiotas can have an impact on dairy calves' growth and development (65, 66). The gut microbiota exists symbiotically with the host by stimulating the immune system and intestinal tissue development while obtaining energy from the host's diet (67). Calves' health depends on a healthy intestinal microbiota, which contributes to a stable intestinal environment (17) that promotes epithelial cell growth and development (68).

Pre-weaning calves get most of their energy and protein from partially digested and absorbed food elements in the small intestines (69). Diet has a significant impact on the development of intestinal microbiota (65, 70) and is regarded as one of the most essential elements in gut microbial structure regulation (71–73). This is especially important later in life, as the early intestinal microbiota improves health and productivity (43). As a result, low incidences of illnesses in calves fed PWM compared to raw SM (1, 74) could be attributed to the intestinal microbiota being regulated prior to weaning (52). However, the long-term effects of feeding raw WM on calf health and performance are yet to be elucidated. Interestingly, there were no differences in the alpha diversity of colon and caecum digesta in calves fed raw WM, PWM and raw SM as determined by Chao 1 and Shannon indices (75). However, there were differences in the dominant microbiota in the colon and caecum digesta. In caecum digesta samples, the dominant bacterial phyla in raw WM and PWM-fed calf were *Bacteroidetes* spp. and *Firmicutes* spp., while the dominant bacterial phylum in raw SM calf was *Fusobacteria* spp. In colon digesta samples, *Bacteroidetes* spp. and *Firmicutes* spp. were the dominant phyla in the PWM calves, whereas *Proteobacteria* spp was the most abundant phyla in the raw WM and SM calves. Meanwhile, the study also indicated that feeding calves PWM elevated the relative abundance of *Ruminococcus* spp., *Megamonas* spp., and *Oxalobacter* spp. in the caecum. Notably, *Ruminococcus* spp. produce short-chain fatty acids, which are important sources of energy for ruminants (76). These findings implied that feeding calves PWM may exert beneficial effects on calf health by elevating the abundance of beneficial bacteria in the caecum. Calves fed SM increased the relative abundance of the genus *Comamonas* spp. in the colon (76), which is associated with the degradation of steroids and various aromatic acids (77). This means that the hydrolytic and fermentative functions of raw SM in the colon may have been enhanced. The combination of MR and butyric acid can increase the abundance of *Prevotella* spp. in the colon and cecum (78). *Prevotella* spp. has been established as an important member of the mammalian gut ecosystem, comprising species capable of fermenting a wide range of non-cellulosic plant polysaccharides and protein (79). Studies have shown a decrease in the relative abundance of *Clostridium* spp. and *Peptostreptococcus* spp. in the gut of ASM and PSM calves (80). A study reported that adding sodium humate and glutamine to MR significantly increased the abundance of calves' intestinal beneficial microbiota (81). The nutritional composition of MR affects the calf gut microbiome. A study found that feeding calves two different commercial MRs had different effects on the calves' gut microbiome (82). In addition to the different crude protein and crude fat content of the two MRs, there were also differences in their production methods. Among them, MR with more conjugated milk oligosaccharides significantly increased the abundance of *Bifidobacterium* spp. and *Faecalibacterium prausnitzii* spp. in the gut of calves (82). *Bifidobacterium* spp. is well known to utilize milk oligosaccharides (83).

### Effect on Antimicrobial Resistance in Dairy Calves

Due to the increased attention toward antimicrobial resistance in recent years, the antimicrobial residues in raw WM have also attracted interest among animal scientists. Studies have shown that if raw WM is fed, the antimicrobial residues in the milk might exert selective pressure on the intestinal microbiota in calves, which may increase the prevalence of drug-resistant bacteria in the rumen and intestines (84). Wray et al. (24) evaluated the effect of antibiotic-containing WM feeding calves in two trials. Fecal *E. coli* were monitored for antibiotic resistance. They used fermented and unfermented WM in the first trial. According to these researchers, the geometric mean minimum inhibitory concentration (MIC) for streptomycin was considerably greater for isolates from calves given unfermented WM. In the second study, only unfermented milk was utilized, and no changes in the percentage of resistant *E. coli* and *Enterococci* were found between isolates from calves fed antibiotic-containing milk and the controls when it came to identifying the percentage of resistant *E. coli* and *Enterococci* (24). Consistently, it has been reported that fermentation can reduce the antibiotic content in raw WM, and the high numbers of bacteria in the raw WM might have presented a disease risk in these two experiments. Studies that investigated the impact of feeding raw SM or WM—PWM or not—on calf antibiotic resistance of specific fecal bacteria, found that the proportion of resistant *E. coli* isolates was significantly higher in calves fed raw WM (inclusive of PWM; most pronounced for cephalosporins) than in calves receiving raw SM (15). This implies that PWM represents an acceptable feed for young calves (5). Maynou et al. (15, 38) studied the antimicrobial resistance patterns in fecal *E. coli*, and nasal *Pasteurella multocida* (*P. multocida*) isolates from calves fed either MR or raw WM in 8 commercial dairy farms (4 farms per feeding program). These authors reported a greater number of fecal *E. coli* resistant to enrofloxacin, florfenicol, and streptomycin. Moreover, multidrug-resistant *E. coli* phenotypes were isolated in the feces of calves fed raw WM than in those fed MR (15). Hence, feeding calves raw WM fosters resistant bacteria in the lower gut and respiratory tract of dairy calves. Moreover, feeding raw WM increases the prevalence of pathogenic microbiota resistant to antimicrobials and other non-antimicrobial treatments used on farms (15, 38). Furthermore, feeding raw WM to dairy calves increased the prevalence of antimicrobial-resistant bacteria on the farms as well as other antibiotics that had never been used before (5, 14). Consistently, a study reported that bacterial isolates from human, animal and environmental sources shared genetically identical plasmids that mediate resistance to various antimicrobial classes (85). Although fecal shedding is influenced by a variety of factors, such as the environment and calf age, feeding milk with antimicrobial residues contributes significantly to an increased prevalence of antimicrobial-resistant (AMR) bacteria, such as extended-spectrum beta-lactamase (ESBL)-producing *E. coli*. A study assessed the impact of feeding raw WM on the prevalence of these bacteria in the feces of calves and found an increase in the prevalence of resistant bacteria shedding in the feces (25). Several other studies have reported that feeding raw WM to calves could change their microbiome (86). Consequently, feeding raw WM might lead to poor health by reducing intestinal microbiota diversity (87–89).

Raw WM may also contain non-antimicrobial drugs, such as non-steroidal anti-inflammatory drugs (NSAIDs) used to treat cows with pain and inflammation (90). In addition to variations in the level of antimicrobial residues, it should be noted that raw WM may vary in its composition from one cow to another and farm to farm. A longitudinal study investigated the presence of *E. coli* that produces broad-spectrum-lactamase (ESBL) on a single farm with 250 Holstein-Friesian cows and 40 un-weaned calves (25). All calves were fed raw WM from sick dairy cows or freshly calved cows that had previously received antibiotic dry cow treatment (DCT). In this study, three raw WM samples contained cefquinome and cephalexin residues. Two of the three raw WM samples also contained CTX-M-producing *E.coli*. Moreover, ten calves were individually assessed for the excretion of CTX-M ESBL and found that the excretion time of these AMR pathogens took as long as 64 days (median of 36 days) after the calf had been weaned. Meanwhile, the frequency of excretion of resistant bacteria decreases with time (91). In the first weeks of dairy calves' life, the excretion of resistant bacteria increases, followed by a subsequent reduction (92). Furthermore, the development of *E. coli* resistance in feces and *P. multocida* in nasal samples of calves was documented when fed MR compared to raw WM (15). In the feces, calves that received raw WM reported a higher occurrence of resistant and multi-resistant *E. coli* bacteria. The group fed raw WM had a greater proportion of resistant *P. multocida* to colistin in nasal swab samples. Overall, it was shown that giving raw WM to animals increased the prevalence of antimicrobial-resistant bacteria (15). Much of the antibiotics used on dairy farms are for disease management in mature cows, and AMR in their fecal microorganisms is relatively rare. However, the feces from young dairy calves tend to contain high levels of antimicrobial resistance genes of *E. coli* and *Salmonella enterica*, which may provide dairy farms with potential hosts for antimicrobial resistance genes (93). The antimicrobial resistance in calf feces may be mainly from raw WM feeding. The presence of multidrug-resistant *E. coli* in raw WM urges the need for on-farm practices, such as pasteurization, that reduce the risk of exposure to calves and dissemination of resistant bacteria into the environment (94). A study documented that calves were fed raw SM from birth to weaning, with half of the calves receiving milk spiked with four antibiotics, and the other half fed the same raw SM but without the addition of any antimicrobials. The results showed a significant increase in the proportion of fecal shedding of *E. coli* resistant to ceftiofur as well as several multidrug-resistant bacteria (14). Limited information on antimicrobial resistance to ASM and PSM is available. The effects of raw WM and PWM on calf intestinal microbiota and antimicrobial resistance were summarized in Table 2.

**Table 2:** Raw WM and PWM effect on the intestinal microbiota and antimicrobial resistance of calves.

**Feeding type**	**Intestinal microbiota**	**Antimicrobial resistance**	**References**
WM	Decreased alpha diversity of pre-weaning calf fecal microbiota and decreased abundance of beneficial gut bacteria. Increase in potential pathogens such as *Campylobacter* spp., *Pseudomonas* spp., and *Chlamydophila* spp.	A higher risk of AMR in pathogens	(95, 96)
WM	Increase of the relative abundance of *Acetitomaculum* spp.	Increased risk of AMR	(2, 97)
WM	Influence relative abundance of microbial cell functions, especially with genes linked with stress response, regulation and cell signaling, and nitrogen metabolism.	Increased prevalence of AMR bacteria, such as ESBL-producing *E.coli*	(98, 99)
WM	*Prevotella* spp. was the only dominant genus in the WM calves.	Feeding WM selects ESBL bacteria in calves, and the majority of ESBL isolates (93%) were co-resistant to aminoglycosides	(75, 100)
WM	Decreased the abundance of *Prevotella* spp.	Increased risk of AMR	(2, 93)
PWM	Increased the abundance of *Faecalibacterium* spp. and *Bacteroides* spp.	The proportion of resistant *E. coli* isolates was significantly lower in calves fed PWM than in calves receiving WM	(5, 101)
PWM	Increased the abundance of the *Prevotella* spp*., Faecalibacterium* spp., and *Bacteroides* spp.	Reduce pathogens	(75, 102)
PWM	Increased the abundance of *Prevotella* spp.	Reduce resistant *E. coli*	(5, 103)
PWM	Increase inhibition of *E. coli* and *Staphylococcus aureus*	Reduce the risk of AMR	(104, 105)
PWM	Reduced *Salmonella* spp.	Increases the rate of AMR	(95, 106)

*Pasteurized waste milk (PWM); Waste milk (WM); Antimicrobial resistance (AMR); Extended-spectrum beta-lactamase (ESBL)*.

### Risks of Feeding Raw WM Having Pathogenic Microorganisms on Calf Health

Limiting intestinal microbiota diversity leads to poor gut health (88, 89), which has far-reaching consequences on the development of the host immune system (87). It has been documented that an inflammatory reaction might occur in the jejunum and ileum in calves fed either untreated or acidified raw WM (75). However, calves fed raw WM had much higher risks of getting diarrhea than calves fed treated (i.e., pasteurized or acidified) WM in a recent study (e.g., body condition score, nasal discharge, the occurrence of diarrhea) (20). However, some studies have shown that feeding raw WM to calves can reduce diarrhea (107).

The effects of feeding MR both with and without antimicrobials have been well documented in previous studies (108, 109). A study found that feeding a low cocktail of antimicrobials in MR led to a shift in the bacterial taxa of the intestinal microbiome, including a reduction in *E. coli*, which might positively affect calf health by reducing the occurrence of diarrhea in young calves (110). Pereira and colleagues studied the functional profile of fecal bacteria till 6 weeks of age in calves fed milk with or without antimicrobial residues (98). After 1 week, the two feeding groups showed a significant difference in the abundance of genes in fecal bacteria for stress response, nitrogen metabolism, regulation, and cell signaling.

It has been reported that adding antibiotics to feed can alter the composition of certain microbiota, such as *E. coli* in calves (111, 112). *E. coli* is the most common facultative anaerobe in human and animal gastrointestinal tracts, causing a number of diseases such as diarrhea (113). Calves fed raw WM had reduced relative abundance of *Clostridium* spp. and *Streptococcus* spp., resulting in changes at the genus level (75). This implies that feeding raw WM may reduce calf diarrhea caused by *Clostridium* spp. and *Streptococcus* spp. The potential effect of feeding raw WM to calves on drug resistance has not been studied deeply because the selection of resistant bacteria has traditionally been assumed to occur at concentrations between the susceptible wild-type population's minimal inhibitory (MIC) and the resistant bacteria's MIC. Antimicrobial medications at concentrations below the MIC (sub-MIC) have been shown in other investigations to increase mutagenesis and recombination, resulting in bacterial adaptability to diverse stresses, including antimicrobial pressure (114, 115). An increase in mutagenesis can also result in a heterogeneous increase in the MIC of the bacteria across a range of antimicrobials.

In a controlled study, antibiotics in medicated MR have reported lower morbidity compared to those fed with non-medicated MR (11). The pre-weaning modulation of the intestinal and fecal microbiota may be responsible for this reduction (14). The utilization of discarded milk as calf feed has always been a source of contention. Increased environmental hygiene or WM's role as a vector for several infections has been the main concern (43). Since the establishment of antimicrobial resistance has accelerated in recent years (116), greater attention has been focused on the antibiotic residue concentration in raw WM. According to one theory, the intestinal microbiota of calves given raw WM may be subjected to selection pressure as a result of such residues. This could lead to an increase in the predominance of resistant bacteria in the calf's intestines. Although a few studies have looked into this topic, more results are required for validation. The main controversy surrounding raw WM feeding has been around the high load of pathogenic microbiota (10), especially coliform bacteria that can result in infection in calves (117). There remain controversies over whether raw WM should be used in calf feeding programs, especially due to the presence of pathogenic bacteria (11, 118). High coliform counts in raw WM could lead to high endotoxin levels, which might cause harm to neonatal calves (117). Several species such as *Streptococcus* spp., *Enterobacteriaceae* spp., *Staphylococcus* spp., and *E. coli* have been isolated from raw WM (5). *E.coli* is a major risk for diarrhea during calves' first week of life. Thus PWM would be the best choice to decrease the risk of these harmful bacteria in calves. It has been reported that ASM can inhibit the activity of *Salmonella* spp. by reducing the pH value (119).

Although raw WM may contain many harmful pathogens, however, PWM can kill the harmful bacteria, hence reducing the risk of spreading infectious diseases through feeding raw WM. Despite several studies exploring this question, their results are inconclusive (100). Pasteurization of WM inactivates bacteria, such as the destruction of *Mycobacterium paratuberculosis, spp. Salmonella* spp. and *Mycoplasma* spp. (12, 120, 121), most viruses, such as the bovine leukemia virus (122) and protozoa, such as Cryptosporidium parvum oocysts (123).

## Effect on the Metabolism of Calves

**Figure 2:**
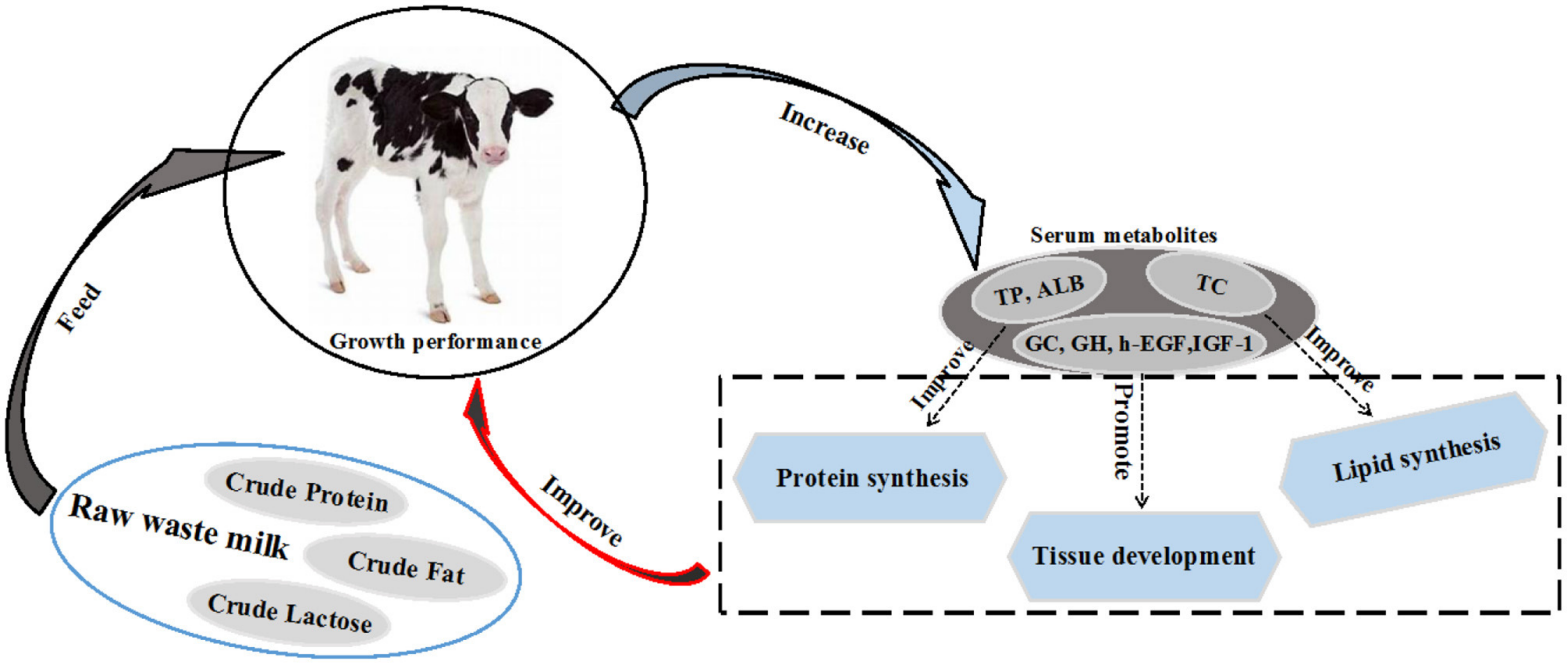
Raw waste milk improves growth performance by increasing serum metabolites total protein (TP), albumin (ALB), total cholesterol (TC), glucagon (GC), growth hormone (GH), human epidermal growth factor (h-EGF) and insulin-like growth factor-1 (IGF-1).

The profile of serum metabolites is a good indicator of the health and nutritional performance of the calf. Among the most important blood parameters, total protein (TP) is a good indicator of health in the first few weeks of life. Twenty-four hours after birth, calves are considered to have a failed passive immunity transfer when their serum TP is less than 5.2 g/dL (64). Furthermore, the Brix value is another important indicator for evaluating the passive immunity of calves. It is generally believed that when the Brix value is lower than 7.8% or 8.4%, calves have failed the passive transfer immunity (124, 125). Besides TP, albumin (ALB) and blood urea nitrogen (BUN) reflect protein utilization (126), while triglycerides (TG) and total cholesterol (TC) reflect the lipid metabolism (127) in young calves. The serum TP decreases when calf feed intake is reduced, there are nutritional imbalances in the diet or failure of passive immunity (124, 128). A previous study reported that the serum TP, ALB, high-density lipoprotein cholesterol (HDL-C), TG and TC concentrations of raw WM fed calves were higher than those in calves fed raw SM (23). This implies that raw WM can improve protein and lipid synthesis in dairy calves (129). In the same study, calves fed raw WM had a greater concentration of growth hormone and glucocorticoids (GC) compared to calves fed raw SM (22). This may signify that calves fed with raw WM had better growth performance (Figure 2). Some studies reported that the retinol metabolic pathway of calves in the MR group was significantly up-regulated compared with the raw SM group (31). Studies have shown that retinol metabolism is related to feeding efficiency (130). This suggests that MR may have a positive effect on calf feeding efficiency. However, it has been reported that MR down-regulates the fatty acid synthesis pathway (104). Notably, the nutrient composition of different MRs varies widely, and more studies are needed to assess the effects of different nutrient compositions of MRs on calf metabolism. The research on the effects of raw SM, WM, PWM and MR on the metabolism of calves is limited, and hence, more in-depth studies are warranted to determine the impact of raw WM and PWM on the metabolic performance of calves in order to provide more practical feeding guidelines. The effects of raw WM and PWM on calf metabolism were shown in Table 3.

**Table 3:** Effect of raw WM and PWM on the metabolism of calves.

**Feeding type**	**Metabolism**	**References**
WM	Increased the serum TP, ALB, HDL-C, TG and TC concentrations	(23)
WM	Improve protein and lipid synthesis in dairy calves	(129)
WM	Improved the concentration of growth hormone (GH) and glucocorticoids	(22)
WM	Increased the serum urea nitrogen concentration	(35)
WM	Increased the serum GH and insulin-like growth factor	(131)
PWM	No effect on calf serum β-hydroxybutyrate concentration	(37)
PWM	No serum glucose changes were found	(37)
PWM	Decreased the serum glucose concentration	(132)

*Pasteurized waste milk (PWM); Waste milk (WM); Growth hormone (GH); Total protein (TP); Albumin (ALB); Total cholesterol (TC); High-density lipoprotein cholesterol (HDL-C); Triglycerides (TG)*.

## Effect on Antioxidant and Immunity of Calves

### Effect on Antioxidant of Calves

The antioxidant system in mammals can protect against the harmful effects of free oxygen species and their metabolites. The system is normally in a dynamic state of balance between free radical generation and clearance, ensuring the animal's optimal health. Calves continue to build their antioxidative defense system after birth (133). Copper and zinc superoxide dismutase are found in milk (134, 135). One of the most common enzymes used to assess oxidative state in animals is superoxide dismutase (SOD) (136, 137). A study reported no difference in the concentration of serum SOD between calves fed raw SM, raw WM and PWM (23). Glutathione peroxidase GSH-px converts superoxide to water (138). A study reported that the serum GSH-px concentration of calves fed with raw SM was significantly higher than calves fed with PWM (38). Furthermore, the level of serum malondialdehyde (MDA) is used to monitor lipid peroxidation via reactive oxygen species (139), representing the degree of lipid peroxidation as well as the degree of free radical damage. Studies have demonstrated higher MDA in serum from calves fed raw SM compared to raw WM and PWM (23). These studies implied that raw SM could activate the antioxidant mechanisms of SOD and GSH-px, while lowering the oxidation mechanisms by MDA production. The results further suggested that high-quality nutrients and microbial activity in raw SM contributed to establishing antioxidant defense mechanisms in calves. However, few dairy farms feed calves with SM due to the associated production costs.

Intrinsic components of the complement system include C3 and C4. Thus the immune system can only function properly if the levels of C3 and C4 are maintained within given ranges. However, compared to raw WM and raw SM, no effect was noted on the content of C3 and C4 when PWM was fed to preweaning calves (23). This means that feeding PWM had no adverse effects on the immune system of the calves. On the other hand, serum immunoglobulin (Igs) levels can directly reflect the regulatory mechanism of humoral immunity. For example, a study showed that the serum IgG content in calves with diarrhea was significantly lower than that of healthy calves and the serum IgG concentration was positively correlated with the incidences of diarrhea (140). A study reported that the IgG content in the serum of calves fed with WM was lower than that of calves fed SM (23). More harmful microorganisms might have caused contributed to the observed results, further buttressing the assertion that direct feeding of raw WM poses a threat to the health of the calf. The effects of raw WM and PWM on calf antioxidant were shown in Table 4.

**Table 4:** Effect of raw WM and PWM on the antioxidant and immunity parameters in calves.

**Feeding type**	**Antioxidant**	**Immunity**	**References**
WM	No effect on calf serum SOD and MDA concentration	Decreased the serum IgG concentration and increased the serum IgA concentration	(23)
WM	Decreased the serum MDA concentration	No effect on calf serum *IL-1β* and *TNF-α*. Increased the jejunal *IL-10* expression	(23)
PWM	No effect on calf serum SOD concentration	Increased the serum IgM concentration	(23)
PWM	Decreased the serum GSH-px concentration	No effect on calf serum IgM concentration	(15, 23)
PWM	Decreased the serum MDA concentration	Decreased the jejunal *IL-10* expression	(23)

*Pasteurized waste milk (PWM); Waste milk (WM)*.

### Regulation of Serum Immune Factors

When the body experiences inflammation, interleukins, types of immunological factors, are activated and regulate the immune system. Interleukins including IL-6, IL-8, and IL-10 are inflammatory markers (141, 142). Tumor necrosis factor (TNF) is engaged in cell-mediated immune responses and plays a key role in intracellular viral and mycoplasmal resistance and defense (143, 144). It has been reported that calves fed with raw WM showed up-regulation of some of the immune factors (e.g., serum IL) in serum, jejunal mucosa and mesenteric lymph nodes (145). This might be linked to the microbial activity of different microbial species and quantities. A study reported that compared with ASM, PSM significantly increased calf serum TNF-α, IL-6 and IL-1β (146). The effects of raw WM and PWM on calf immunity were shown in Table 4.

## Recommendations for Better Use of WM Under Practical Conditions

### Effect of Various Raw WM Treatments on Its Quality

As bacteria can be transmitted directly from cows (cows with mastitis) to milk, poor hygiene during milking, transportation, and milk storage often exacerbates contamination. Raw WM that has not been properly treated should be used cautiously to feed calves, as it has a greater microbial load that can be harmful to calves. The geometric mean of the cumulative number of bacteria for raw WM samples was shown to be significantly higher than in raw SM; *Streptococcus* spp. and *Enterobacteriaceae* spp. were the predominant bacteria identified, followed by *Staphylococcus* spp. (10). Milk pasteurization is currently used on many dairy farms to minimize the potential risk of infectious diseases in calves (147). It has been documented that PWM could reduce the morbidity and mortality of calves and increase their growth rate compared to those fed conventional MR (9). Dairy producers and ranchers use the pasteurization process to kill off a large number of the pathogenic microorganisms in milk, such as *Mycobacterium avium subsp. paratuberculosis* (148–150) and *Salmonella* spp. or *Mycoplasma* spp. (10, 121). Milk pasteurization can be accomplished by heating the raw WM to 63°C for 30 min (low temperature, long time) or 72°C for 15 s (high temperature, short time) (5). A study reported that pasteurization of raw WM at 63°C for 35 min could increase phenotypic resistance to ampicillin, cephalotin, ceftiofur, and florfenicol in fecal *E. coli* (38). Although, Grant et al. (151) suggested that pasteurization of raw WM with a longer holding period was more successful in inactivating harmful bacteria, more research has shown that optimal raw WM pasteurization conditions were attained at 63°C for 30 min (19, 152). Compared with raw WM or MR, feeding PWM can ensure better growth performance and health status and obtain higher economic profits (9, 153).

Acidifying WM has also been recognized as a labor-saving, simple and cost-effective method in calf feeding operations (154). Consequently, the method can reduce the rapid growth of pathogens in the digestive tract and decrease the incidences of infectious bacteria (155).

Although raw WM can be pasteurized to inactivate bacteria (12, 120, 156), the method might not be effective on spore-forming bacteria (157), some viruses and protozoan (158). For example, *M. avium* spp. cannot completely be inactivated by pasteurization (159–161). Aust et al. (5) reported the importance of pasteurization and its link to a decrease in the threat of transmission of *Streptococcus agalactiae, Mycobacterium paratuberculosis, E. coli* and *Mycoplasma* spp. Notably, certain bacterial toxins (117) and residual antibiotic concentrations may not respond to pasteurization (162).

### Recommendations for the Use of Raw WM as a Feed Source in Calves

The health status of the dairy calves should be prioritized when deciding whether to feed raw WM. Firstly, cows with BVDV or Johne's disease can easily spread the diseases to other animals and calves. Thus, it would be better to avoid feeding milk from such cows to calves. Secondly, the WM should not be kept at room temperature for an extended period since this may cause major changes in microbial load. Thirdly, the milk obtained from the first milking soon after antibiotic use should be avoided because this milk is a major source of antibiotic residues (163). Moreover, Feeding calves raw WM from cows infected with *E. coli* or *Pasteurella* spp. should also be avoided because these bacteria stay in the milk for a long time and can likely invade the intestinal barrier, contributing to the calf sickness. Collectively, it is recommended that the colostrum obtained from cows must be free from diseases like enzootic bovine leukosis, tuberculosis and brucellosis. Similarly, colostrum from cows showing any sign of infectious disease at the time of milking should not be used. Pasteurization conditions and effects on calf health are shown in Table 5.

**Table 5:** Pasteurization condition and improvement of raw WM.

**Feeding type**	**Pasteurization Condition**	**Improvement**	**References**
PWM	72°C for 15 s	Increased the feed efficiency	(23)
PWM	72°C for 15 s	The potential benefits of pasteurization in disease prevention outweigh the potential risks of feeding a non-pasteurized WM	(16)
PWM	73.5°C for 20–25 s	Decreased the *Mycobacterium avium subsp* of WM	(164)
PWM	63°C for 30 min or at 72°C for 15 s	Inactivates bacteria	(5)
PWM	72–74°C for 16 s	Not show significant negative effects on the intake, ruminal parameters, blood parameters, health, or performance of dairy calves.	(37)
PWM	63°C for 35 min	Increased the presence of phenotypic resistance to ampicillin, cephalotin, ceftiofur, and florfenicol in fecal *E. coli*	(38)
PWM	63°C for 30 min	Decreased the total bacteria in waste milk	(152)
PWM	62.7°C for 30 min or 71.6°C for 15 s	Pasteurization can be very effective in lowering bacterial contamination of milk	(19)
PWM	65.5°C for 30 min	Destroy *M. paratuberculosis* in WM	(165)
PWM	65°C for 10 min	Destroyed common mastitic mycoplasma such as *Mycoplasma bovis, M. californicum*, and *M. canadense*	(121)

*Pasteurized waste milk (PWM)*.

## Conclusions

Altogether, we concluded that raw WM is economical and contributes positively to the growth and performance of dairy calves. However, the antibiotic residues and the presence of some pathogenic bacteria, such as *Mycobacterium avium subsp. Paratuberculosis, Salmonella* spp. and *Mycoplasma* spp., may limit the use of raw WM as a feed resource in calves. Consuming WM results in the shedding of antibiotic-resistant bacteria in the feces of calves. Feeding properly pasteurized WM can decrease bacterial load in the milk and reduce the risk of diseases in calves. However, it is advisable to avoid feeding raw WM obtained from cows that have been treated with antibiotics over a long time during lactation.

## Author Contributions

YM, MK, and ZC: conceptualization and writing—original draft preparation. YM, MK, JX, GA, XC, SL, YW, and ZC: editing and technical review. YM, MK, JX, GA, and XC: Searching for literatures. ZC: visualization and supervision. All authors have read and agreed to the published version of the manuscript.

## Funding

The review was supported by the China Animal Husbandry Group (DR201905) and the 2115 Talent Development Program of China Agricultural University. The funders had no role in the study design, data collection, analysis, decision to publish, and manuscript preparation.

## Conflict of Interest

The authors declare that the research was conducted in the absence of any commercial or financial relationships that could be construed as a potential conflict of interest.

## Publisher's Note

All claims expressed in this article are solely those of the authors and do not necessarily represent those of their affiliated organizations, or those of the publisher, the editors and the reviewers. Any product that may be evaluated in this article, or claim that may be made by its manufacturer, is not guaranteed or endorsed by the publisher.
